# Recent Lifestyle Parameters Are Associated with Increasing Caesarean Section Rates among Singleton Term Births in Austria

**DOI:** 10.3390/ijerph16010014

**Published:** 2018-12-21

**Authors:** Sylvia Kirchengast, Beda Hartmann

**Affiliations:** 1 Department of Anthropology, University of Vienna, A-1090 Vienna, Austria; 2 Clinic for Gynaecology and Obstetrics, Danube Hospital, A-1220 Vienna, Austria; beda.hartmann@wienkav.at

**Keywords:** caesarean section rates, delivery mode, recent environment, maternal age, maternal height, obesity, newborn size, macrosomia, child presentation

## Abstract

Caesarean section (CS) rates are increasing in many parts of the world, recently reaching about 20% worldwide. The postmodern lifestyle characteristics, obesity and delayed childbirth, have been put forward as the main reasons for high CS rates. The present study tests the association patterns between lifestyle parameters and delivery mode on a data set of 3786 births in Vienna between 2005 and 2013. The focus is exclusively on singleton term births. As well as maternal age, prepregnancy weight status, maternal body height and gestational weight gain, newborn size (birth weight, birth length, and head circumference), Apgar scores and child presentation were recorded. Planned as well as emergency CS rates increased significantly (*p* < 0.0001) with increasing maternal age and decreasing maternal body height. Emergency CS rates, however, increased significantly with increasing maternal prepregnancy weight status and gestational weight gain. An especially high risk of emergency CS occurred among four groups of mothers: those older than 40 years (OR = 2.68; 95% CI 1.87–3.86), those who were obese (OR = 1.44; 95% 1.15–1.81), those experiencing a gestational weight gain above 15 kg (OR = 1.32; 95% CI 1.13–1.54), and those shorter than 160 cm (OR = 1.216; 95% CI 1.02–1.45). Emergency CS rates were significantly higher among low-weight newborns (<2500 g) and macrosome newborns (>4000 g) than among normal-weight newborns. Furthermore, breech presentation was associated with an increased risk of caesarean delivery (OR 6.97; 95% CI 6.09–7.96). Logistic regression analyses reveal that maternal age, maternal body height, prepregnancy weight status, gestational weight gain, birth weight, newborn head circumference and child presentation show an independent, highly significant association with caesarean delivery. We conclude that maternal and newborn characteristics typical of recent lifestyle patterns, such as advanced maternal age, obesity, increased gestational weight gain and increased newborn size, are highly significantly associated with increased emergency CS rates. Moreover, maternal shortness and breech presentation are risk factors for emergency CS.

## 1. Introduction

Today, caesarean sections are among the most frequently performed surgeries on women, and caesarean delivery rates continue to rise worldwide [1,2]. Clearly, caesarean section (CS) is a life-saving surgical procedure that has helped to decrease maternal and neonatal mortality rates dramatically during the 20th century [3,4]. Up to the early 20th century, CS was performed mainly on dying or already dead parturient because the severe bleeding and infections associated with the surgical procedure resulted in a maternal death rate of almost 100% [3]. During the 20th century, CS became increasingly safe and the related maternal mortality dropped markedly [5]. Despite this clearly positive effect, the worldwide rise in CS rates has become a growing public health concern and a cause for considerable debate due to potential maternal and perinatal risks, cost issues and inequity in access [6,7,8,9,10]. This growing debate is mainly due to the 1985 World Health Organization statement that “regional caesarean section rates should not exceed 10 to 15%” [11]. According to recent estimates, the prevalence of CS, however, is much higher. 

Currently, the average global rate of CS is about 18.6%, although the prevalence differs considerably between different regions [2,12]. As expected, the lowest CS rates (<3%) occur among low-income countries, such as in western Africa. In contrast, extraordinarily high CS rates are documented for the Dominican Republic (56.4%) and Brazil (55.6%), but also in Egypt (51.8%), Iran (47.9%), Turkey (47.5%) and Italy (38.1%) [2]. In most European countries, the United States and New Zealand, the rates are about 25 to 35% [2]. These values are clearly higher than the WHO recommendations. Although the validity of this WHO threshold (10–15%) has been increasingly questioned in recent years, analyses of the factors behind the increasing CS rates and the development of strategies to reduce these rates have gained importance [7]. This trend of questioning the justification of caesarean deliveries is mainly due to their extremely high rates and the associated immediate and long-term risks [13,14,15,16]. 

Various reasons for these high CS rates have been discussed. On the one hand, obstetricians recommend CS because in their opinion it is a safe surgical technique with many benefits for the fetus, such as a reduced risk of trauma, hypoxic encephalopathy and cerebral damage caused by prolonged hypoxic status [17]. Furthermore, CS may reduce the risk of operative vaginal delivery and damage to the pelvic floor [17]. Consequently, CS is considered to be less risky than vaginal births. On the other hand, CS upon maternal request has gained in importance [18,19,20,21,22]. The present study, however, excludes this aspect. 

So-called evolutionary medicine provides a completely different approach to explain rising CS rates. Besides evolutionary factors such as bipedalism and encephalization, which have complicated human birth [23], a mismatch between recent lifestyle patterns and the environment in which we evolved may have increased birth complications, resulting in increasing CS rates [24]. This goes beyond the mismatch between the environment of our general adaption and recent life circumstances as, during the last few decades, our lifestyle has drastically changed. This change apparently also affects birth and the mode of delivery [25]. A typical lifestyle characteristic that potentially affects the delivery mode is the postponement of first reproduction up to the fourth or fifth decade of life. This trend has been described for most OECD countries, with the reported increase of age at first reproduction ranging from 2 to 5 years between 1970 and 2015 [26]. These changing reproductive patterns mainly reflect marked changes in our social environment. On the one hand, higher education and participation in the labor force have given women greater economic independence. On the other hand, access to abortion and high-quality contraceptives have made it much easier for women to choose the optimal time of reproduction. Advanced maternal age, however, is often associated with an increased risk of CS [27,28], but also with other risk factors of caesarean delivery including obesity, hypertensive diseases and diabetes [29,30]. Obesity, however, is an age-independent risk factor for complications during pregnancy and birth [31]. During the last few decades, overweight and obesity rates have increased steadily in both high-income and in middle- and low-income countries [32]. A high intake of energy-rich food in combination with reduced physical activity, both behaviors typical of our recent environment, results in high rates of overweight people and obesity. In 2008, for the first time in human history, the number of overweight people worldwide exceeded that of undernourished people [33]. Especially among women of reproductive age, this trend towards increasing obesity rates has fatal consequences [34,35,36,37]. Obesity per se is a risk factor of caesarean delivery [38,39,40,41]. Furthermore, maternal obesity increases the risk of giving birth to macrosome or large-for-gestational-age (LGA) newborns. Macrosomia, however, also increases the risk for higher caesarean section rates [42]. The present study analyzes the impact of two important recent lifestyle factors on the delivery mode among healthy mothers in Vienna, Austria. Specifically, we test the hypothesis that postponing reproduction and obesity increase the rate of emergency caesarean sections.

## 2. Material and Methods

### 2.1. Data Set

The present retrospective study analyzes a data set of 3786 singleton births that took place at the Danube Hospital (SMZ Ost) in Vienna, Austria (A1220) between 2005 and 2013. The Danube Hospital is one of the largest public birth clinics in Austria. During the study period, a total of 17,430 births were recorded here. Only 3786 births are incorporated in the present investigation. This restriction mainly reflects the strict inclusion and exclusion criteria. For example, all prenatal medical examinations had to be carried out at the Danube Hospital. Furthermore, only healthy primiparae mothers of Austrian or Central European origin who experienced a term delivery (39th and 40th week of gestation) of a single infant without congenital malformations were enrolled. Additional exclusion criteria included registered maternal diseases such as HIV infection, diabetes mellitus or nephropathy before and during pregnancy, hypertension (BP < 150/90 mmHg), preclampsia, drug or alcohol abuse, and any type of medically assisted reproduction. This process yielded the final set of 3786 recorded births. Pre- and postnatal care is highly developed in Austria. All Austrian residents have social insurance that covers all medical costs in public hospitals. During the 1970s, the so-called “mother–child-passport” system was introduced. This sophisticated system of pre- and postnatal care comprises seven check-ups during pregnancy starting at the eighth week of gestation and includes eight postnatal check-ups of the child between birth and the fourth year of life. Pregnant women are required to do all the check-ups, which are free of charge. Prenatal examinations are performed in consulting rooms of gynecologists or at the clinic where birth is scheduled to take place. Postnatal examinations are performed in pediatrician consulting rooms. The introduction of this “mother–child-passport” helped to reduce the neonatal and child mortality rate dramatically in Austria during the 1970s. All data collected at the individual checkups are documented at the hospital and in the above passport, which belongs to the mother. Complete mother–child-passports are rewarded with a financial premium by the government. Each prenatal check-up includes sonographic investigations of the fetus and documents maternal health, diseases, smoking behavior and weight gain. After birth, the delivery mode, complications, duration of delivery, newborn size and Apgar scores are documented. The present study analyzes the data of 3786 primiparae women between the ages 18 and 48 years (x = 28.3 ± 5.4) at the time of first birth and their newborns. All women enrolled in the present study belonged to the Viennese middle class and had regular social insurance. The medical treatment at the Danube Hospital was covered by social insurance and none of the enrolled women required private insurance.

### 2.2. Maternal Parameters

Besides family status and nicotine consumption during pregnancy, the following maternal somatometric parameters were collected at the first prenatal visit (eighth week of gestation): body height, prepregnancy weight, and weight at the end of pregnancy. Body height was measured to the nearest 0.5 cm using a standard anthropometer at the first prenatal visit. All women were asked to report their body weight before pregnancy. Additionally, body weight was measured to the nearest 0.1 kg on a balance beam scale. Based on the literature, the first 13 weeks of gestation involve an extremely small weight gain of only 1.7% [43]. Consequently, prepregnancy weight was calculated as the mean value of the reported weight and the weight at the eighth week of gestation. Weight at the end of pregnancy was measured before birth. The gestational weight gain was calculated by subtracting prepregnancy weight from body weight at the end of pregnancy. Weight status was determined by means of the body mass index (BMI) kg/m^2^. To classify maternal weight status, we used the cutoffs published by the WHO [44]. A BMI below 18.50 kg/m^2^ was classified as underweight. Normal weight was defined as a BMI between 18.50 and 24.99 kg/m^2^, overweight was defined as a BMI between 25.00 and 29.99 kg/m^2^. A BMI above 30.00 kg/m^2^ was classified as obese. Gestational age was calculated in terms of the number of weeks from the beginning of the last menstrual bleeding to the date of delivery (=duration of amenorrhea) and by two consecutive ultrasound examinations performed before the 12th week of gestation. 

### 2.3. Newborn Parameters

The following parameters were taken directly from the newborns, immediately after birth: birth weight in grams using a digital infant scale, birth length in centimeters using a standard measurement board for infants and head circumference in centimeters using a tape. Low birth weight (LBW) was defined as a birth weight below 2500 g, and macrosomia as a birth weight above 4000 g according to the WHO recommendations [45]. 

### 2.4. Apgar Score

To evaluate the newborn vital functions, the one-, five- and ten-minute APGAR scores were used. The Apgar score was introduced in 1952 as a simple and repeatable method to assess the health status of newborns immediately after birth. Five simple criteria, skin color/complexion, pulse rate, reflex irritability, muscle tone and breathing, are evaluated using a scale from zero to ten. The Apgar scoring system remains as relevant for predicting neonatal survival today as it was 60 years ago [46].

### 2.5. Obstetrical Characteristics

The following obstetric characteristics were recorded: delivery mode, i.e., spontaneous vaginal delivery, vaginal operative delivery (forceps, vacuum extraction), planned caesarean section and emergency caesarian section. All planned caesarean sections were carried out exclusively for medical reasons. In the present study, the main reasons for planned caesarean sections were cephalo-pelvic disproportion (diagnosed by sonography), adverse child presentation or placenta previa. Caesarean sections upon maternal request without any medical indication were not performed at the Danube Hospital. The most frequent indications for emergency caesarian delivery were fetal distress and obstructed labor. The intra-uterine position of the infant at the time of delivery (head presentation, pelvic presentation, and transverse presentation) was also included in the analysis.

### 2.6. Statistical Analysis

Statistical analyses were carried out using SPSS for Windows (version 24.00, IBM, Austria). The Kolmogorov–Smirnov test indicated the normal distribution of most metric variables. Therefore, parametric tests were performed exclusively. After computing descriptive statistics, Duncan analyses with Bonferroni corrections and χ^2^ were calculated to test the differences between spontaneous vaginal deliveries, operative vaginal deliveries, planned caesarean sections and emergency caesarean sections. Odds ratios were calculated to analyze the risk of experiencing emergency CS among obese mothers, short mothers, mothers experiencing high gestational weight gain, macrosomia, low birth weight and breech presentation. Additionally, binary logistic regression analyses were performed to evaluate maternal as well as newborn factors associated with caesarean section. *p* < 0.05 was considered statistically significant. 

## 3. Results

### 3.1. Sample Characteristics

Table 1 presents maternal age and somatic characteristics, as well as information regarding family status and smoking behavior. Most women gave birth for the first time between the ages of 20 and 29 years; the mean age at first birth, however, was 28.3 years. More than 85% of the mothers did not smoke during pregnancy. Less than 25% of the women enrolled in the present study were classified as short (<160 cm), only 5.8% were tall (>175 cm). More than 65% of the women corresponded to the definition of normal weight before pregnancy. In total, 18.1% were classified as overweight, and 9.2% as obese. Gestational weight gain was high. The highest value was 52 kg. More than 40% of the women experienced a gestational weight gain of more than 15 kg. Only 7% gained less than 7 kg during pregnancy. One woman lost 8.2 kg during pregnancy. Newborn somatometrics and Apgar scores one, five and ten minutes after birth are presented in Table 2. Data concerning newborn weight status revealed that 90.4% of the newborns corresponded to the definition of normal weight. Only 1.6% were classified as low weight (<2500 g), while 8.0% were classified as macrosome, i.e., their birth weight exceeded 4000 g. The prevalence of breech presentation was 5.9%.

### 3.2. Delivery Mode

**Figure 1:**
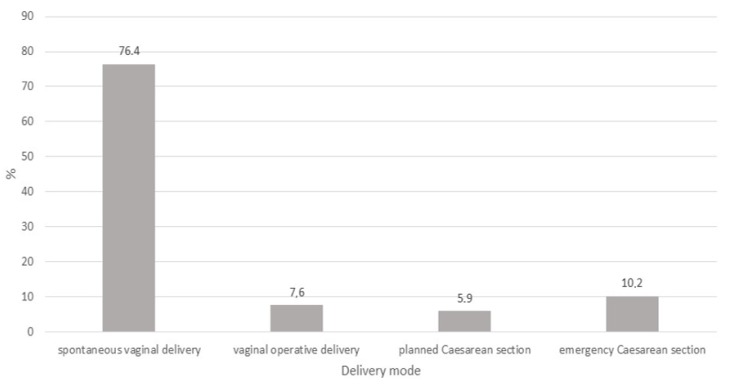
Percentage of delivery modes.

More than 75% of the enrolled women experienced a spontaneous vaginal delivery. Forceps or vacuum extraction was performed among 7.6% of the mothers, and CS was performed among 16.1% of the mothers. In total, 5.9% of the deliveries were planned CS, while 10.2% of the deliveries required emergency CS (Figure 1).

### 3.3. Maternal and Offspring Factors Associated with Delivery Mode

Maternal as well as newborn characteristics differed significantly between the four delivery modes. As presented in Table 3, women experiencing spontaneous vaginal deliveries were significantly younger than those experiencing caesarean section or operative vaginal delivery. Women delivering through emergency CS were significantly the shortest, heaviest and showed the significantly highest gestational weight gains. In contrast, the newborns delivered by emergency CS were significantly larger and heavier than newborns delivered vaginally or by planned caesarean section. Apgar scores were significantly highest among newborns delivered by spontaneous vaginal delivery. The prevalence of emergency CS increased significantly with maternal age and weight. It was most frequent among women gaining more than 15 kg during pregnancy. With increasing body height, however, the prevalence of emergency CS decreased significantly (Table 4). 

In the next step, risk factors for emergency caesarean section were analyzed. In general, the risk of emergency CS was significantly increased among mothers older than 40 years versus those who were younger than 40 years, among obese mothers versus normal weight mothers, among women gaining more than 15 kg during pregnancy versus those gaining less than 15 kg, and among women shorter than 160 cm versus those taller than 160 cm. Concerning newborn size, CS rates were significantly highest among LBW newborns (22.6%). Among macrosome newborns, CS rates (14.5%) were significantly higher than among normal weight newborns. Low birth weight as well as macrosomia increased the risk of emergency CS significantly in comparison to normal weight newborns. Furthermore, breech presentation increased the risk of emergency CS highly significantly in comparison to head presentation. (Table 5).

The results of the ANOVA and chi-square analyses were corroborated by binary logistic regression analyses. Both spontaneous vaginal delivery and operative vaginal delivery were defined as vaginal delivery. Both planned and emergency caesarean sections were defined as caesarean section. While marital status and birth length had no significant impact on delivery mode, caesarean section was significantly positively associated with nicotine consumption, maternal age, prepregnancy weight status, gestational weight gain, breech presentation, birth weight and newborn head circumference. Maternal body height, in contrast, was negatively associated with CS (Table 6).

## 4. Discussion

Caesarean section is defined as “the surgical termination of pregnancy or delivery by operative opening of the uterus”. Today, CS is recommended when vaginal delivery might pose a risk to the mother or the fetus. Nonetheless, the recently extraordinarily high CS rates are not explained solely by increased risk or medical indications [18]. It is well documented that caesarean deliveries are recommended by obstetricians as a very safe type of delivery [17,47] and that they are increasingly requested by pregnant women [20]. Accordingly, high CS rates may be explained by this trend towards elective caesarean deliveries. This raises the question of whether high CS rates are caused only by this trend, or do they reflect lifestyle changes that might increase emergency CS too? Liston [24] tried to explain rising CS rates by evolutionary as well as environmental factors. In fact, during the 20th and 21st century, human lifestyles have changed dramatically, especially over the last few decades. Rapid urbanization, a drastic decrease of physical activity and the rising prevalence of obesity and associated diseases typify this transition [48]. Furthermore, reproductive patterns, mainly in developed countries, have undergone major change. The mean number of offspring has decreased, and the age at first birth has increased. Some of these lifestyle changes have a profound impact on human birth patterns. The present study analyzes the impact of recent key lifestyle factors, in particular obesity and delayed childbirth, on the mode of delivery. The hypothesis is that prepregnancy obesity and an increased maternal age increase the risk of emergency caesarean section.

From a medical viewpoint, the main reasons for CS (excluding CS upon maternal request) are obstructed labor, twin pregnancy, high blood pressure of the mother, transverse or breech presentation or fetal distress [49]. The present study excludes twin pregnancies and maternal hypertension; no transverse presentation was documented. The rate of breech presentation was 5.9%. As expected, breech presentation resulted mainly in planned CS (80.7%) and emergency CS (18.4%). 

The main focus of the present study was on the maternal and fetal factors potentially associated with the delivery mode. In the present sample, the CS rate was 16.1%. Of these, 5.9% of the births were planned CS, mainly caused by cephalo-pelvic disproportion; 10.2% were classified as emergency CS, i.e., where a vaginal delivery was initially planned but a medical indication for caesarean delivery developed during the birth process. These CS rates found in the present study were markedly lower than the CS rates in Austria during the study period between 2005 and 2013. During this period, the CS rate in Austria increased from 25.5% to 29.3% [50]. In 2017, the Austrian CS rate reached 29.7% [50]. The lower prevalence of CS in the present study reflects two factors: CS upon maternal request is not performed at the Danube Hospital, and we applied strict inclusion and exclusion criteria. Only singleton term births of healthy mothers were included. In order to focus exclusively on healthy mothers, women suffering from diseases such as diabetes mellitus or hypertension were excluded, although we are aware that both diabetes mellitus and hypertension may be associated with overweight and obesity, conditions whose association with delivery mode are addressed here. Nevertheless, the present analysis focused on healthy mothers exclusively. Specifically, we compared spontaneous vaginal deliveries, operative vaginal deliveries (forceps or vacuum extraction), planned CS and emergency CS. The results show that with increasing maternal age, the prevalence of spontaneous vaginal deliveries decreased significantly. Women experiencing planned as well as emergency caesarean deliveries were significantly older than those experiencing vaginal deliveries. Maternal age was an important predictor of caesarean delivery. In the present sample, the emergency CS rate among mothers older than 40 years was 23.2%. This was true only of 7% of mothers younger than 20 years. In total, 8.5% of mothers aged 20 to 29 years and 12.5% of those aged 30 to 39 years experienced emergency CS. As we included only primiparae women, the conclusion is that delaying childbirth increases the risk of CS, above all emergency CS. Several previous studies document a significant association between advanced maternal age (>35 years) and an increased likelihood of CS [28,51]. This association may be interpreted as a result of a changing social environment. In recent decades, the age at first birth has increased continuously in most industrialized countries. In Austria, for example, the value increased from 23.8 years in 1984 to 29.5 in 2017 [50]. In particular, the number of mothers older than 40 years nearly doubled in Austria from 2.6% in 2004 to 4.11% in 2017 [50]. This trend of delaying childbirth and the increasing number of older mothers increases CS rates too.

Besides the effects of advanced maternal age on emergency CS rates, we also focused on maternal obesity as a risk factor. The body mass index of women experiencing emergency CS was significantly higher than that of women experiencing vaginal deliveries or planned CS. Furthermore, women experiencing emergency CS gained significantly more weight during the gestational period than women experiencing vaginal deliveries or planned CS. Obese women showed the highest rates of emergency CS. These results support several previous studies. It is well documented that obese women are more likely to face induction of labor, CS, but also anesthetic problems, wound infections and postpartum hemorrhage [30,37,52]. Prepregnancy obesity is mentioned as one of the most important maternal risk factors for caesarean delivery [31,39,40,41,53,54,55]. This is especially true of morbid prepregnancy obesity. According to an Australian study, morbidly obese mothers, i.e., with a body mass index above 50 kg/m^2^, have a significantly higher risk of obstetric complications during pregnancy and birth, and 51.6% of these super-obese women gave birth via caesarean section [56]. As pointed out above, changes in our lifestyle such as reduced physical activity in combination with high calorie diets not only increase obesity rates but also have detrimental effects on female reproductive outcome. Currently, more than 50% of women aged 20 to 39 years are overweight or obese in the United States. Similar patterns are reported for Europe, where one in five pregnant women can be classified as obese [57,58]. Obese pregnant women are confronted with a four-fold increased risk of developing gestational diabetes (GDM), which may result in fetal macrosomia and thus increase CS rates as well. 

Macrosomia, i.e., a birth weight above 4000 g, is the main cause of obstructed labor, after fetal–pelvic disproportion. Macrosomia is mainly associated with maternal obesity and maternal diabetes but also advanced maternal age [42]. In the present study, 14.5% of macrosome newborns were delivered by emergency CS, and only 4.5% by planned CS. Typical recent lifestyle patterns, such as postponing reproduction up to the fourth or even fifth decade of female life and high obesity rates, increase the risk of macrosome newborns and large head circumferences of newborns. Both macrosomia and large head circumferences increase the risk of CS. The present study clearly underlines these positive associations between newborn size, especially birth weight and head circumference, and increased CS rates. A detailed analysis of the impact of fetal growth patterns, in particular head dimensions and child presentation on delivery mode, is in preparation. Nevertheless, we already show here that recent lifestyle patterns characterized by delaying childbirth and obesity promote the development of maternal and newborn characteristics that increase the risk of CS. 

During the 20th century, people have not only become heavier but they have also become taller [59,60]. Increasing body height, however, has a positive effect on delivery mode. Maternal shortness and low body height are recognized obstetric risk factors because short maternal height may be associated with cephalo-pelvic disproportion (CPD), resulting in obstructed labor [61]. Maternal shortness therefore represents an important risk factor for emergency CS [62,63,64]. Witter et al. [65] showed that a maternal height of less than 157 cm was significantly associated with an increased risk of CS, whereby cephalo-pelvic disproportion (CPD) and labor arrest have been mentioned as the main causes for emergency CS in short women [61,66,67,68]. The present study supports previous observations that maternal height significantly influences delivery mode. Our results show that maternal height is negatively related with the risk of CS. This risk increases significantly with decreasing maternal height. In contrast, tall women (>175 cm) showed the lowest rate of emergency CS. Unfortunately, the secular trend in body height growth is declining in almost all European countries. In recent decades, body height has tended to stabilize, whereas body weight continues to grow. Overweight people and obesity are taking pandemic forms in developed countries [59]. Therefore, we should not expect a continuing trend towards increased tallness, which might reduce emergency CS rates. In contrast, increasing rates of obesity and increasing age at first birth, both associated with a higher prevalence of macrosome newborns, may well increase CS rates. 

## 5. Conclusions

Caesarean section is no doubt a life-saving surgical procedure that has helped to dramatically reduce maternal and neonatal mortality rates. Nevertheless, CS rates of 30% and more are clearly too high. The present study verifies the hypothesis that recent lifestyle characteristics, such as postponement of reproduction and obesity during the reproductive phase, increase emergency caesarean sections. Increasing obesity rates, high gestational weight gain and advanced maternal age increase both the prevalence of macrosome newborns and emergency CS rates. 

## Figures and Tables

**Table 1:** Maternal sample characteristics, absolute and relative frequencies.

Maternal Characteristics	Mean (SD)	Range	*n* (%)
**Civil status**			
Unmarried			1795 (47.4%)
Married			1882 (49.7%)
No information			109 (2.9%)
**Nicotine consumption during pregnancy**			
Yes			558 (14.7%)
No			3228 (85.3%)
**Maternal age**	28.3 (5.4)	18–48	
**Maternal age group**			
<20 years			100 (2.6%)
20–29 years			2050 (59.4%)
30–39 years			1380 (36.4%)
≥40 years			56 (1.5%)
**Stature height (cm)**	165.9 (6.3)	148.1–189.0	
<160 cm			871 (23.0%)
160–165 cm			1013 (26.7%)
166–175 cm			1681 (44.4%)
>175 cm			221 (5.8%)
**Prepregnancy weight (kg)**	63.9 (13.3)	44.0–150.1	
**End of pregnancy weight (kg)**	78.5 (13.9)	48.6–55.0	
**Pregnancy weight gain**	14.7 (5.7)	−8.2–52.1	
<7 kg			189 (5.0)
7–15 kg			2064 (54.5)
>15 kg			1533 (40.5)
**Prepregnancy Body mass index (kg/m^2^)**			
Underweight <18.50			269 (7.1%)
Normal weight 18.50–24.99			2480 (65.5%)
Overweight 25.00–29.99			685 (18.1%)
I = Body mass index Obese > 30.00			352 (9.3%)

**Table 2:** Newborn sample characteristics, absolute and relative frequencies.

Newborn Characteristics	Mean (SD)	Range	*n* (%)
**Sex**			
Female			1878 (49.6%)
Male			1908 (50.4%)
**Birth weight (g)**	3383.7 (427.5)	1745–5110	
**Newborn weight status**			
Low birth weight <2500 g			62 (1.6%)
Normal weight 2500–3999 g			3421 (90.4%)
macrosome ≥4000 g			303 (8.0%)
**Birth length (cm)**	50.7 (1.9)	37.0–58.0	
**Head circumference (cm)**	34.2 (1.3)	29.0–43.0	
**Apgar 1 min**	9.1 (1.2)	0–10	
**Apgar 5 min**	9.8 (0.7)	0–10	
**Apgar 10 min**	9.9 (0.5)	0–10	
**Child presentation**			
Head presentation			3563 (94.1%)
Breech presentation			223 (5.9%)

**Table 3:** Maternal and newborn characteristics according to delivery mode (ANOVA, Bonferroni post hoc test).

Maternal and Offspring Factors	Spontaneous Vaginal Birth	Vaginal Birth Operative	Planned CS	Emergency CS	*p*-Value
	Mean (SD)	Mean (SD)	Mean (SD)	Mean (SD)	
**Maternal Factors**					
Age (years)	27.9 (5.2) ^bcd^	29.2 (5.3) ^a^	30.0 (5.6) ^a^	29.6 (5.7) ^a^	0.001
Body height (cm)	166.1 (6.3) ^d^	165.2 (6.2)	166.5 (6.2) ^d^	164.6 (5.9) ^ac^	0.001
PPW (kg)	63.5 (12.8) ^d^	64.2 (14.5)	64.9 (13.3)	66.6 (16.1) ^a^	0.001
PPBMI (kg/m^2^)	23.02 (4.37) ^d^	23.44 (4.84) ^d^	23.37 (4.51) ^d^	24.52 (5.41) ^abc^	0.001
EPW (kg)	77.9 (13.4) ^d^	78.8 (14.2) ^d^	79.9 (13.7)	82.2 (16.2) ^a,b^	0.001
GWG (kg)	14.5 (5.6) ^d^	14.7 (6.7)	15.2 (5.8)	15.4 (5.9) ^a^	0.042
**Offspring Factors**					
Birth weight (g)	3381.4 (414.8) ^cd^	3440.4 (434.6) ^c^	3238.4 (416.1) ^abd^	3445.3 (496.4) ^ac^	0.001
Birth length (cm)	50.7 (1.9) ^cd^	50.9 (2.2) ^c^	50.2 (1.8) ^abd^	51.1 (2.3) ^ac^	0.001
HC (cm)	34.1 (1.3) ^bcd^	34.4 (1.4) ^bc^	34.7 (1.3) ^ab^	34.6 (1.3) ^a^	0.001
Apgar 1 min	9.3 (0.9) ^bcd^	8.5 (1.4) ^ac^	8.9 (1.0) ^abd^	8.3 (1.9) ^ac^	0.001
Apgar 5 min	9.9 (0.6) ^bcd^	9.5 (0.9) ^ac^	9.7 (0.6) ^abd^	9.4 (1.2) ^ac^	0.001
Apgar 10 min	9.9 (0.5) ^bd^	9.8 (0.8) ^ac^	9.9 (0.3) ^bd^	9.8 (0.9) ^ac^	0.002

Legend: PPW = prepregnancy weight; EPW = end of pregnancy weight; PPBMI = prepregnancy body mass index; GWG = gestational weight gain; HC = head circumference. ^a^ = significantly different from spontaneous vaginal birth; ^b^ = significantly different from vaginal birth operative; ^c^ = significantly different from planned CS, ^d^ = significantly different from emergency CS.

**Table 4:** Maternal and newborn characteristics according to delivery mode, χ^2−^ analyses.

Maternal and Newborn Characteristics	Spontaneous Vaginal Delivery	Vaginal Delivery Operative	Planned CS	Emergency CS	*p*-Value
**Marital status**					
married	75.5%	8.4%	5.8%	10.1%	0.15
unmarried	77.7%	6.5%	5.8%	9.9%	
**Nicotine consumption**					
no	76.1%	7.9%	6.1%	10.0%	0.130
yes	78.0%	5.6%	5.0%	11.5%	
**Maternal age group**					
<20 years	83.0%	7.0%	3.0%	7.0%	0.0001
20–29 years	79.5%	6.9%	5.0%	8.5%	
30–39 years	71.8%	8.5%	7.3%	12.5%	
≥40 years	51.8%	12.5%	12.5%	23.2%	
**Prepregnancy weight status**					
underweight	80.1%	8.2%	5.2%	6.4%	0.003
normal weight	78.0%	7.2%	5.8%	9.1%	
overweight	72.1%	8.2%	6.7%	12.9%	
obese	71.1%	8.0%	5.7%	15.2%	
**Gestational weight gain**					
<7 kg	73.5%	9.7%	5.9%	10.8%	0.029
7–15 kg	78.4%	7.4%	5.4%	8.8%	
>15 kg	73.9%	7.9%	6.1%	12.1%	
**Maternal body height**					
<160 cm	73.8%	8.3%	5.1%	12.9%	0.002
160–165 cm	75.2%	7.9%	5.5%	11.4%	
166–175 cm	77.7%	7.2%	6.3%	8.9%	
>175 cm	81.9%	5.9%	8.1%	4.1%	
**Newborn weight status**					
SGA < 2500 g	64.5%	4.8%	8.1%	22.6%	0.0001
2500–4000 g	77.4%	7.3%	6.1%	9.2%	
LGA > 4000 g	67.7%	10.6%	4.5%	14.5%	
**Child presentation**					
head presentation	82.8%	7.1%	1.2%	9.1%	0.0001
breech presentation	0.4%	0.4%	80.7%	18.4%	

**Table 5:** Caesarean section risk factors.

Parameter	Odds Ratio	95% Confidence Interval
Maternal age < 40 years	2.68	1.87–3.86
Prepregnancy BMI > 30.00 kg/m^2^	1.44	1.15–1.81
Gestational weight > 15 kg	1.32	1.13–1.54
Body height < 160 cm	1.22	1.02–1.45
LBW newborn < 2500 g	2.02	1.37–2.95
Macrosome newborn > 4000 g	1.42	1.13–1.78
Breech presentation	6.97	6.09–7.96

**Table 6:** Maternal and newborn characteristics and delivery mode. Vaginal delivery versus caesarean section. Binary logistic regression analysis.

Maternal and Newborn Characteristics	Coefficient	SE	Significance	Exp(B)	95% CI
	Dependent variable: delivery mode (VD = 1; CS = 2)				
Marital status	0.082	0.115	0.477	1.09	0.866–1.359
Nicotine consumption	0.362	0.165	0.028	1.437	1.040–1.985
Maternal age	0.075	0.010	<0.0001	1.079	1.056–1.103
Maternal body height	−0.056	0.009	<0.0001	0.945	0.928–0.963
Prepregnancy BMI	0.082	0.011	<0.0001	1.085	1.062–1.110
Gestational weight gain	0.049	0.010	<0.0001	1.050	1.029–1.072
Birth weight	0.001	0.001	0.005	1.048	0.999–1.049
Birth length	0.081	0.047	0.083	1.085	0.994–1.190
Head circumference	0.288	0.056	<0.0001	1.334	1.195–1.489
Child presentation	2.195	0.103	<0.0001	8.982	7.303–11.046
